# Barriers to airway procurement processes in sub-Saharan Africa: An exploratory qualitative study

**DOI:** 10.4102/jcmsa.v4i1.257

**Published:** 2026-03-13

**Authors:** Nina R. Patel, Fiona Kabagenyi, Felicia Tshite, Isaac Barnor, Tagwa Abdalla, Reuel Maina, Josh Wiedermann, Lia Jacobson, Samuel Okerosi, Karthik Balakrishnan, Rolvix H. Patterson, Douglas Sidell, Mary Jue Xu, Taseer Din

**Affiliations:** 1 Department of Otolaryngology, University of San Francisco, San Francisco, United States of America; 2 Department of Ear Nose and Throat, Faculty of Medicine, Makerere University, Kampala, Uganda; 3 Department of Otorhinolaryngology, Faculty of Medicine, University of Pretoria, Pretoria, South Africa; 4 Department of Otolaryngology, Faculty of Medicine, St Peters Hospital, Jacobu, Ghana; 5 Department of Ear Nose and Throat, Faculty of Medicine, University of Khartoum, Khartoum, Sudan; 6 Department of Ear Nose and Throat, Kenyatta National Hospital, Nairobi, Kenya; 7 Department of Otolaryngology, Mayo Clinic Rochester, Rochester, United States of America; 8 Department of Otolaryngology, Stanford Medicine, Palo Alto, United States of America; 9 Department of Head and Neck Surgery and Communication Sciences, Duke University Health System, Durham, United States of America; 10 Department of Otolaryngology, Sidra Medicine, Doha, Qatar; 11 Department of Otolaryngology, Weill Cornell University Hospital, Doha, Qatar

**Keywords:** airway equipment procurement, sub-Saharan Africa, otolaryngology, emergency airway management, medical supply chains, healthcare infrastructure, qualitative study, stakeholder perspectives, health systems strengthening

## Abstract

**Background:**

Access to high-quality airway equipment is critical for effective otolaryngological and emergency care. In sub-Saharan Africa (SSA), challenges in the procurement infrastructure may hinder equipment availability, impacting patient outcomes. Given the paucity of literature investigating equipment procurement in SSA, this study aims to map out the multilevel barriers to procurement of airway equipment in SSA to inform opportunities for improvement.

**Methods:**

A qualitative study design was employed using semi-structured interviews and open-ended survey responses. Participants included otolaryngologists in SSA, manufacturing company representatives and international policymakers, selected through purposive sampling to ensure representation of various roles in the procurement process. Six interviews, one focus group and six survey responses with regional healthcare providers (*n* = 13), manufacturing representatives (*n* = 3) and international policymakers (*n* = 2) explored supply chain dynamics, regulatory challenges, manufacturing capabilities, financial constraints and policy frameworks. Data were analysed using thematic analysis to identify recurrent themes and interrelationships.

**Results:**

Systemic barriers exist in the procurement process, including fragmented supply chains, inconsistent regulatory enforcement, limited local manufacturing capacity and insufficient funding mechanisms. Otolaryngologists emphasised frequent delays in equipment availability, while manufacturers reported logistical challenges and regulatory hurdles. Policymakers noted gaps in infrastructure and regional coordination. Emerging themes suggest opportunities for streamlining procurement processes, fostering local production and enhancing collaboration to improve supply chain efficiency.

**Conclusion:**

This study underscores the multifaceted challenges in airway equipment procurement in SSA and highlights the need for integrated approaches involving stakeholders across the supply chain.

**Contribution:**

Findings provide actionable insights to inform policy development and capacity-building initiatives with the goal of enhancing access to life-saving airway equipment and improving healthcare outcomes.

## Introduction

The Lancet Commission on Global Surgery has highlighted the critical role of access to essential surgical care and equipment in improving health outcomes worldwide. It estimates that over 5 billion people lack access to safe, timely and affordable surgical and anaesthesia care, disproportionately affecting populations in low- and middle-income countries (LMICs). In these settings, surgical and anaesthesia care remains significantly underdeveloped, with large gaps in infrastructure and equipment availability.^1^ Among the essential components of surgical and emergency care, airway management has been cited as a priority area that requires highly specialised equipment to ensure patient survival in critical, time-sensitive situations.^2,3^ Emergency airway management is a critical component of acute surgical care and is essential for treating life-threatening conditions such as airway obstruction and respiratory failure. The World Health Organization (WHO) and the Disease Control Priorities Project (DCP3) recognise its role in reducing perioperative mortality and strengthening emergency care systems.^4^

Despite growth in the global otolaryngology workforce and advancements in airway management protocols, many LMICs and sub-Saharan Africa (SSA) in particular continue to face critical barriers to accessing essential equipment for safe and effective airway intervention.^5^ Recent literature has identified SSA as the region reporting the highest rates of unavailable or insufficient airway management equipment, including rigid bronchoscopes and endoscopic towers.^6^ These shortages are driven by a complex interplay of financial constraints, supply chain disruptions, limited local manufacturing and regulatory hurdles.^5^ Another cross-sectional study of paediatric airway equipment availability further highlighted the particular scarcity in SSA, where 55% of surveyed hospitals lacked access to rigid bronchoscopes, severely limiting the capacity for life-saving interventions.^7^

The lack of paediatric airway equipment is especially consequential given the time-sensitive nature of airway emergencies in children (such as foreign body aspiration, subglottic stenosis or severe airway trauma), which require immediate intervention to prevent irreversible hypoxic injury or death. In a commentary by Kabagenyi et al., eight specialists from seven African countries estimated that each hospital sees an average of 40 patients per month requiring critical surgical airway management, with roughly 2–25 preventable deaths per year in each country attributable to lack of access to essential equipment for airway foreign body removal.^8^ Another study in Ethiopia documented an annual incidence rate of 2.26 per 100 000 children for paediatric airway foreign body aspiration.^9^ Together, these findings underscore the urgent need for targeted investments in paediatric airway infrastructure, equipment procurement and provider training to reduce preventable morbidity and mortality from airway foreign bodies in SSA.

This study aims to investigate the barriers limiting access to airway equipment in SSA to inform actionable solutions that improve access to essential equipment for airway management. By engaging with clinicians, policymakers and medical device manufacturers, this research provides critical insights into how systemic, structural, economic and logistical factors impact the availability and accessibility of airway equipment and outlines potential strategies for intervention.

## Research methods and design

### Study design

This qualitative study maps multilevel barriers within the procurement infrastructure and processes governing access to emergency airway equipment in SSA. Given the multiple players and components involved in manufacturing, distributing and using airway equipment within the broader context of public policy, the team chose a qualitative study design to provide more in-depth and robust data on system-level processes and multilevel barriers. This approach allowed the research team to capture nuanced perspectives across different stakeholder groups and explore the complex interactions and systemic challenges influencing airway equipment availability in SSA.

### Participant selection

Participants included regional otolaryngologists from SSA (*n* = 13, across eight countries), manufacturing representatives from companies producing or distributing airway equipment in SSA (*n* = 3), and international policymakers (*n* = 2) involved in emergency health policy. Given the complexity of paediatric airway care and the unique challenges it presents, the study specifically included purposive sampling of the PENTAfrica team, a group of clinicians with dedicated subspecialty paediatric airway training, to ensure the study reflected expert, contextually informed perspectives while maintaining regional diversity across SSA.

### Data collection

The interview guide was designed by the study team (Nina R. Patel, Mary Jue Xu, Taseer Din, and Fiona Kabagenyi), which included clinician-researchers with training in otolaryngology, global health and qualitative research methods, and was iteratively refined to reflect the perspectives of multiple stakeholder groups. Semi-structured interviews were conducted from January 2024 to December 2024 using an interview guide questionnaire (Appendix 1 Table 1-A1) tailored to each stakeholder group. The guide explored the following themes: barriers to airway equipment procurement and distribution, existing procurement frameworks and their effectiveness, interactions and coordination among stakeholders, perceptions of local manufacturing capacity and scalability and recommendations for improving airway equipment availability and access. Interviews were conducted in English via either the Zoom video conferencing platform or in person by Nina R. Patel, a researcher in the medical field with prior experience in qualitative interviewing and familiarity with airway management and health systems in low-resource settings. Taseer Din and Fiona Kabagenyi are paediatric otolaryngologists with direct clinical experience caring for paediatric patients with airway pathology, which informed the study design and interpretation of findings. The interviewer did not have supervisory or financial relationships with participants. Each interview lasted approximately 30 min – 60 min and was audio-recorded. Audio recordings were transcribed verbatim by Nina R. Patel, and transcripts were reviewed for accuracy by the research team (Taseer Din and Fiona Kabagenyi).

### Data analysis

An initial coding framework was developed by Nina R. Patel based on the interview guide and refined iteratively through inductive coding. One researcher completed initial transcript coding (Nina R. Patel), and second reviewer consensus was conducted by Taseer Din and Fiona Kabagenyi to ensure reliability and discrepancies were resolved through discussion. Dedoose software was used to manage and organise the data. Additional codebook refinement was completed by Nina R. Patel and Taseer Din during consensus. Thematic analysis focused on identifying common patterns, differences and unique insights across and between stakeholder groups. Interviews were conducted until thematic saturation was reached.

Interview transcripts were analysed using thematic analysis following an iterative, inductive–deductive approach. An initial coding framework was developed by Nina R. Patel based on the interview guide and key sensitising concepts relevant to airway equipment procurement and health systems and was subsequently refined through inductive coding as new concepts emerged from the data.

One researcher completed initial transcript coding (Nina R. Patel) using line-by-line coding of all transcripts, and second reviewer consensus was conducted by Taseer Din and Fiona Kabagenyi through independent review of coded transcripts to ensure reliability, and discrepancies were resolved through discussion with refinement of code definitions. Dedoose software (Dedoose Version 9.2.22, 2025, Los Angeles, California, United States: SocioCultural Research Consultants, LLC, www.dedoose.com) was used to manage and organise the data. Additional codebook refinement was completed by Nina R. Patel and Taseer Din throughout the analytic process to ensure clarity, consistency and conceptual coherence across transcripts.

Thematic analysis focused on identifying common patterns, differences and unique insights across and between stakeholder groups using constant comparison to examine convergence and divergence within and across groups. Codes were grouped into higher-order categories and themes, and interviews were conducted and analysed iteratively until thematic saturation was reached, defined as the point at which no new themes or substantive insights emerged.

### Ethical considerations

Ethical clearance to conduct this study was obtained from the University of California, San Francisco Human Research Protection Program Institutional Review Board (Ref no.: 404673). All participants, including clinicians, trade partners, and policymakers, provided written informed consent prior to participation. To ensure protection of anonymity, all identifying information was removed during transcription and participants were assigned unique identification codes. The study was conducted in accordance with relevant ethical guidelines and regulations governing research involving human subjects.

## Results

**FIGURE 1:**
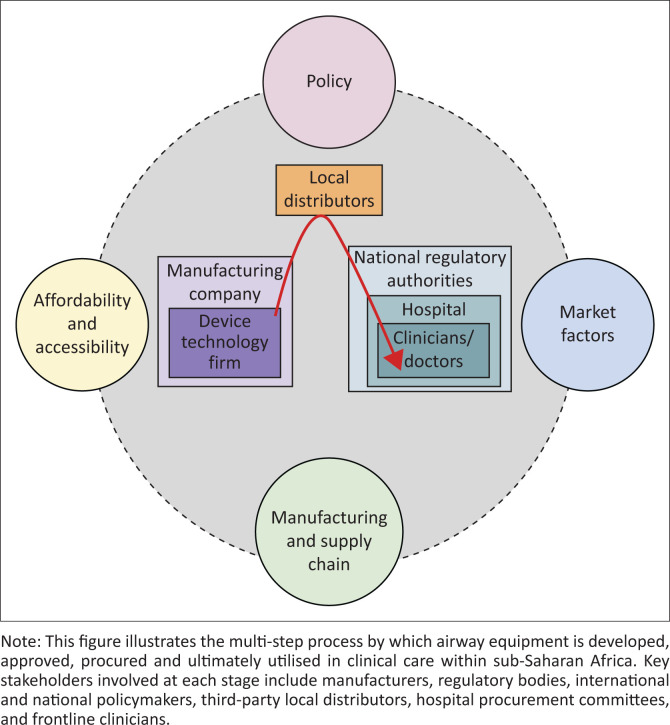
Flowchart of the airway equipment procurement process for sub-Saharan Africa: From technology to clinical use.

The qualitative analysis of six interviews, six participant-based written responses and one focus group with manufacturers, policymakers and clinicians revealed significant barriers and systemic challenges in the procurement and accessibility of airway equipment in SSA. The findings of these qualitative interviews highlight that the procurement of airway equipment in SSA follows a complex, multi-stage pathway from technology development to clinical use. At each stage, systemic factors, including market factors, policy, market dynamics, manufacturing and supply chain constraints and affordability, exert significant influence on stakeholders, shaping access to essential medical equipment. These stakeholders along the entire procurement process include policymakers, hospital administrators, manufacturers, international organisations, and frontline clinicians (Appendix 1 Table 2-A1; Figure 1).

### Procurement processes and key stakeholders

As shown in Figure 1, the procurement processes for airway equipment in SSA are greatly hindered by systemic barriers including uneven access, high costs, inefficient supply chains and fragmented regulatory frameworks.

#### Technology development and manufacturing

Airway equipment originates in research and development, primarily within international medical technology firms. Because of limited local manufacturing capacity, most equipment is produced in higher-income country (HIC) and upper-middle-income country settings, making procurement in regions such as SSA largely dependent on global supply chains and subject to fluctuations in availability and cost. The lack of regional production within SSA increases reliance on imports, leading to delays and higher expenses, as cited by interviewees.

#### Local distributors and profit margins

Once manufactured, equipment is distributed through local distributors, including wholesalers and other intermediary procurement agents (often referred to as ‘middlemen’ by clinicians in the region), whose services increase costs. Clinicians state that these third-party local distributors increase the cost of airway equipment by up to 30%. Market inefficiencies, such as monopolies, lack of regional competition and non-transparent pricing, can further inflate expenses. Hospitals and healthcare systems in SSA often lack direct procurement channels, increasing reliance on third-party vendors to procure equipment.

#### Importation and taxation

Upon arrival in the country, imported equipment is subject to national regulatory approvals, tariffs and customs duties, contributing to further delays and price increases. Policy inconsistencies, lack of regulatory harmonisation and bureaucratic inefficiencies complicate the process, creating barriers to timely distribution. Notably, these fees and taxes vary considerably from country to country, even within the region. Multiple interviewees report being able to bypass these regulations through education-based permits and undisclosed donations. Additionally, limited government subsidies or procurement assistance programmes often leave hospitals struggling to afford necessary equipment. Without centralised funding mechanisms or bulk purchasing agreements, individual hospitals are forced to negotiate prices independently, often at higher rates because of lower purchasing volumes and reliance on third-party distributors. In many cases, government procurement frameworks prioritise pharmaceuticals or larger infrastructure projects, leaving critical yet lower-profile items such as airway equipment chronically underfunded. This financial strain forces hospitals to either delay purchases, rely on emergency procurements at inflated prices or operate with inadequate and ageing equipment, compromising patient care and clinical outcomes.

#### Distribution to hospitals and clinicians

Once imported, equipment is allocated to healthcare facilities based on procurement budgets, donor contributions or private sector investments. Public hospitals, often underfunded, may experience shortages or receive outdated models. Limited supply chain infrastructure and weak maintenance support systems hinder long-term usability, while inadequate funding prevents bulk purchasing, which could otherwise lower costs. At the final stage, clinicians receive the equipment for direct patient care. However, financial constraints, training gaps and inconsistent equipment availability are cited as common challenges by interviewees. Affordability remains a critical issue; hospitals may ration equipment and providers struggle with surgical skill retention because of inconsistent usage. Without proper integration into healthcare systems, even available equipment may go underutilised or remain non-functional. For example, if the equipment is incompatible with existing hospital infrastructure, such as differences in voltage requirements, lack of spare parts or absence of manufacturer support, its utility is significantly diminished. Poor logistical planning, clinician and staff training and inventory management may also lead to essential devices sitting idle in storage rather than being readily accessible in clinical settings. Furthermore, unclear policies on procurement, maintenance responsibilities and user training contribute to inefficiencies that prevent equipment from being effectively incorporated into routine patient care. As a result, this forces many clinicians to purchase their own equipment out of pocket through third-party sites, though there remain no standardised guidelines or processes to regulate quality control of these items.

### Systemic factors

Key metathemes (market factors, manufacturing and supply chain, accessibility and affordability and policy) were each interwoven with themes and subthemes that highlight the overarching influences along the equipment procurement process. Table 1 summarises these codes along with relevant quotes from interviews across policymakers (P), manufacturing representatives (M) and clinicians (C).

**TABLE 1:** Mapping key perspectives: Metathemes, themes and subthemes in airway equipment procurement.

Meta-theme	Theme	Subtheme	Quotes
Market factors	Market coverage	-	‘Most hospitals in urban areas have access to this equipment, but rural areas lack coverage’. (P1) ‘We only distribute to three countries in Sub-Saharan Africa due to logistical challenges’. (M1)
Manufacturing and supply chain	Manufacturing and maintenance capacity	National or Local	‘There’s limited manufacturing capacity within Sub-Saharan Africa, and most equipment is imported’. (M1) ‘Investing in local production facilities could reduce costs significantly’. (P2) ‘Ther’s no facility for repairing broken down stuff. You use it, it works for the first year, and then the next year, you can’t use it anymore’. (C1)
		International	‘Currently manufacturing is only at the international level. This is important because costs are driven up since we are unable to access new equipment without taxes, and fees, and middlemen who cause cost to be more than for those in high income countries. How does that make sense’. (C2) ‘At the level of procurement, there is a problem of knowledge and skill and experience of which equipment is used. Most people who come with great experience, they have been using the big companies like Karl Storz and Philips and et cetera, but there is a bigger market, a global market, which might offer, like, cheaper alternative and more competitive aftercare packages. Why would you buy Storz through government if you’re not negotiating an aftercare plan?’ (C4)
	Supply chain and distribution	National or Local	‘They face significant delays due to inadequate distribution infrastructure’. (P2) ‘Rural areas are especially hard to reach because of poor roads and unreliable transport’. (M2) ‘If I work in a university, I’m getting it for personal use, but I’m going to teach students. For education. This way I am not taxed when equipment enters the country. When the equipment lands in my country, normally I’ll probably pay a withholding tax, which is 6% – 10%’. (C3)
		International	‘Over the years, hundreds of thousands of pounds of equipment have come into Africa through my charity and through, because people have carried it in. And I am told colossal numbers of lies in immigration departments. Only a week ago here, I have a 40 kilogram luggage bag full of stuff. I’ve bought laser arms and all sorts of things. I’ve sent equipment in boxes, but only to my African friends who are going to collect it from the airport or from the shipping authority’. (C5)
	Middlemen	-	‘It’s not just the manufacturers and the hospitals, the entire supply chain is full of brokers and middlemen who each take their cut of up to 30%’. (M1) ‘We have cases where the same equipment would cost double in our country compared to what hospitals in high income countries pay, simply because of the layers of middlemen’. (C6)
Affordability and accessibility	Cost and affordability	-	‘The equipment is too expensive for many public hospitals’. (M3) ‘We need subsidies or grants to make these devices affordable’. (C2) ‘If we were going to say that this is what you need to manage paediatric airway, we have to do that through a guideline development process with PICO questions. And it was a two-year, half-million-dollar activity every time we do it’. (P1)
	Equity and accessibility	-	‘Rural hospitals often struggle to access the same equipment as urban facilities’. (M2) ‘So the idea is to start a registry, and people can donate stuff through, cut out the middleman, because a central space that is validated and that can actually work for everybody to put in things like a virtual sales space like Gumtree. eBay. This provides equity’. (C7) ‘Okay, for my African colleagues… we thought outside the box. So what we do, my colleagues here, we buy the instruments ourselves from India, in Turkey, that’s about 30% of the cost of Karl Storz or Medtronics or whichever other company’. (C8) ‘[*P2*] carries a whole box of instruments for himself, and he walks around different hospitals in the government. The private hospitals have about 95% of the equipment that we need, but most of the hospitals are government, so we buy them for ourselves since residency’. (C9)
Policy	Regulatory and policy environment	International	‘In general, we tend not to go into a lot of depth on specific, very narrow clinical presentations for a number of complex reasons. [*ORG1*] has only really started doing clinical care properly in the last few years, and it was a public health organization. And so, it’s taking some reframing and needs more research to understand’. (P1) ‘You’re all making noise. If someone else is making more noise than you are, it’s because of that. So how do you fix that? The alternative is, if you ask for these things, then probably they say, wait for the next financial year. And you’re waiting for two, three, four financial years’. (C1)
		National	‘Regulatory approval processes in some countries are slow and inconsistent. Variations in withholding tax are based on the whims of local enforcement and government’. (M2)

PICO, population/problem, intervention, comparison, outcome; P, policymakers; M, manufacturing representatives; C, clinicians; ORG1, anonymised organisation.

#### Market factors

An inconsistent market was a challenge from both the perspective of supply and perceived demand.

#### Manufacturing and supply chain

A lack of local manufacturing emerged as a critical barrier. Manufacturers cited high costs, limited infrastructure and regulatory hurdles as possible deterrents to setting up production in the region. Clinicians expressed concerns about inadequate maintenance and aftercare support for existing equipment, with broken devices often sitting unused because of a lack of spare parts and trained biomedical technicians. Policymakers acknowledged that investment in local maintenance services remains underdeveloped and costly, exacerbating the cycle of equipment shortages. Additionally, disruptions among existing supply chains were commonly cited. All stakeholders reported delays caused by poor logistics, inefficiencies in equipment being processed by customs and reliance on external distributors. Manufacturers pointed to weak transportation networks and high import fees, while hospital administrators cited frequent procurement delays, leaving clinicians without necessary equipment for extended periods. Finally, the presence of local distributors, otherwise referred to as ‘middlemen’, in the procurement process significantly inflates costs. Manufacturers explained that third-party distributors and procurement agents add markups at every stage, sometimes doubling or tripling the final price of equipment. Policymakers noted that hospitals often lack direct purchasing channels, increasing dependence on local distributors. Clinicians reported that equipment shortages often result in reliance on emergency procurements, which are more expensive because of local distributor fees.

#### Affordability

Affordability remains a major concern across all stakeholders. Manufacturers stated that high production costs and import fees increase equipment prices. Policymakers cited limited government funding for procurement, thereby forcing hospitals to rely on external funding sources or pass on additional costs to patients, which exacerbates financial barriers to accessing essential airway equipment. Many hospitals, especially in the public sector, struggle to secure financing for necessary devices, often relying on used and potentially broken equipment as a result. The unaffordability of equipment exacerbates disparities in the healthcare system. Airway equipment availability is highly uneven, with urban hospitals having better access than rural or under-resourced regions. Policymakers and clinicians acknowledged disparities across private, governmental and rural settings, while manufacturers expressed concerns that current procurement models fail to incentivise equitable distribution. Finally, clinicians noted that cost barriers not only limit availability but also affect training and surgical skill retention, as inconsistent access prevents providers from maintaining practice and skills in complex airway surgery.

#### Policy

Complex, heterogeneous and unclear regulatory policies for procuring equipment were a major barrier to efficient market engagement and timely distribution. Policymakers acknowledged that regulatory frameworks are often fragmented across countries, making it difficult to streamline approvals and processes. Clinicians and hospital administrators also noted that unclear procurement policies at the national and hospital levels contribute to delays, sometimes leading to the purchase of incompatible or substandard equipment.

### Recommendations and proposed solutions

These identified barriers underscore the need for targeted interventions at the policy, advocacy and supply chain levels to streamline procurement, promote local manufacturing and implement equitable distribution strategies that ensure consistent access to essential airway equipment. The recommendations summarised in Table 2 include developing regional manufacturing hubs, strengthening distribution infrastructure and streamlining regulatory processes. Accessibility and affordability barriers were largely financial, with high equipment costs, limited procurement budgets and insufficient financing options for hospitals. Proposed solutions included bulk purchasing agreements, increased public health funding and hospital-led financing models to improve affordability. Policy barriers included weak enforcement of procurement regulations and a lack of regional coordination. Stakeholders emphasised the importance of strengthening regulatory enforcement, fostering multi-sector collaboration and creating standardised procurement frameworks to improve equipment accessibility and long-term sustainability.

**TABLE 2:** Barriers and targeted recommendations for sustainable airway equipment procurement in sub-Saharan Africa.

Theme	Barriers	Recommendations
Market factors	Limited market size and demand unpredictability.	Conduct market assessments to identify demand and support local industry.
	High dependency on imported equipment.	Manufacturers should invest in local production through incentives and partnerships.
	Limited supplier competition monopolises the market.	Support new market entrants with funding and technical assistance.
Manufacturing and supply chain	Insufficient local manufacturing capacity.	Policymakers should develop policies that encourage regional manufacturing and technology transfer.
	Fragmented distribution networks.	Strengthen procurement planning and supplier coordination with the involvement of policymakers, manufacturing companies and other relevant stakeholders.
	High dependency on middlemen.	Encourage pathways for direct engagement between end users and manufacturers, as well as propose innovative models to cut costs.
	Regulatory delays in product approval and importation.	Manufacturers should consider subsidised costs for life-saving airway equipment in order to promote markets.
		Streamline regulatory approvals and harmonise regional standards.
		Create new supply chains with more affordable, locally produced instruments.^10^
		Revitalise donation-based models in which surgical teams and specialists routinely visit LMIC sites, partnered with industry stakeholders committed to providing high-utility equipment donations.
Affordability and accessibility	High costs of imported airway equipment.	Facilitate bulk purchasing agreements to lower costs.
	Limited government subsidies or procurement budgets.	Establish financing models for equipment procurement.
		Increase public health funding for equipment acquisition.
Policy	Weak enforcement of procurement regulations.	Policymakers should strengthen enforcement mechanisms and accountability.
	Lack of regional coordination in procurement.	Support regional procurement frameworks.
	Limited engagement between stakeholders.	Local providers should advocate for policy changes based on clinical needs.

Note: Please see the full reference list of the article, Patel NR, Kabagenyi F, Tshite F, et al. Barriers to airway procurement processes in sub-Saharan Africa: An exploratory qualitative study. J Coll Med S Afr. 2026;4(1), a257. https://doi.org/10.4102/jcmsa.v4i1.257, for more information.

LMIC, low- and middle-income country.

## Discussion

This study highlights the complex, multi-layered challenges in airway equipment procurement in SSA, revealing considerable barriers at the market, supply chain, financial and regulatory levels. These interconnected obstacles create a system where high costs, limited accessibility and unreliable distribution cycles perpetuate equipment shortages, limiting clinicians’ ability to provide life-saving airway interventions. The procurement process for airway equipment is also shaped by systemic inefficiencies, from manufacturing constraints and local distributors inflating costs to restrictive regulatory policies and limited government funding. Existing policies fail to protect end users, including clinicians and hospitals, allowing local distributors to capitalise on these gaps in procurement systems. Without comprehensive oversight, hospitals face inconsistent pricing and supply chain disruptions, further exacerbating disparities in equipment availability. This fragmented and costly procurement system ultimately transfers financial burdens to patients and their families, who often bear out-of-pocket expenses for essential airway care, thereby increasing healthcare costs and economic hardship.

However, our findings suggest opportunities for multilevel interventions. Addressing these barriers requires coordinated, multi-sectoral efforts from policymakers, manufacturers, new low-cost device developers and healthcare institutions to develop sustainable procurement strategies, enhance local production and maintenance capacity and ensure equitable access to essential airway equipment. Strengthening local manufacturing, improving national procurement policies and fostering partnerships between governments and private organisations help to mitigate these challenges. Cross-disciplinary collaboration is essential; clinicians, manufacturers and policymakers must work together to create transparent, efficient systems that ensure sustainable access to high-quality airway equipment.

Prior commentaries and opinion pieces in the existing literature have broadly discussed the challenges in general surgical equipment access in LMICs, often highlighting fragmented supply chains, affordability constraints and limited cost transparency.^11^ Our findings reinforce these observations but also emphasise the critical role of regulatory inefficiencies and the absence of policies protecting hospitals from inequitable pricing. Additionally, our findings that third-party local distributors inflate the cost of airway equipment by up to 30% are supported by industry data showing distributor mark-ups can range from 20% to 40%, underscoring the significant financial impact of intermediaries on procurement costs in LMIC settings.^12^ Unlike previous research, which often focuses on donor-driven solutions, this study underscores the need for systemic, locally driven procurement models. The potential for bulk-buying and pooled demand strategies, modelled after successful human immunodeficiency virus (HIV) antiretroviral (ART) procurement programmes, is an innovative solution for reducing costs and stabilising supply chains.^13^ Organisations such as KidsOR have explored similar models, but scaling such initiatives to national levels may require collaboration with Ministries of Health (MOHs) and regional hospital networks.^14,15^

Given the systemic nature of these barriers, interventions must target multiple levels. Policymakers should prioritise national procurement strategies that eliminate inefficiencies and reduce dependence on costly imports. Ministries of Health can establish centralised purchasing agreements to negotiate better pricing and ensure fair distribution. Manufacturers could focus on expanding regional production and maintenance networks to reduce reliance on expensive imports. Regional clinicians, as end users, must be actively involved in policy discussions to advocate for their needs and ensure that procurement decisions reflect frontline realities. Breaking down existing silos between these stakeholders is critical to creating a sustainable and equitable equipment pipeline.

This study has several limitations. Firstly, it may not fully capture the perspectives of local distributors and end users in rural settings, where procurement challenges could differ significantly because of varying economic structures and access to sea and road transportation, which directly affect logistics. Similarly, our study did not include sufficient data to explore potential differences between private and public healthcare settings in airway equipment procurement, limiting insights into how sector-specific factors may influence barriers and opportunities. Secondly, given the multiple players and components involved in manufacturing, distributing and using airway equipment within the broader scope of public policy, we elected a qualitative approach to provide in-depth and nuanced data on stakeholder experiences and multilevel barriers. While surveys or quantitative approaches could offer broader generalisability, at this early stage of scoping the landscape qualitative methods allowed us to explore complex interactions and capture contextual insights that would be difficult to quantify. We acknowledge that our relatively limited sample size may have constrained thematic saturation across all stakeholder categories and participant populations, which is an important limitation given the heterogeneity of healthcare systems, economic contexts and logistical challenges across SSA countries.

Further research should explore regional variations and compare procurement pathways between private and public healthcare sectors. Studies investigating pricing models could evaluate current institutional expenditures versus potential cost savings through pooled demand strategies. While our findings provide insight into airway equipment procurement in SSA, they may not be generalisable to LMICs with different regulatory frameworks, market dynamics or equipment types. Future work could include analysing procurement pathways for other essential medical equipment, convening multi-stakeholder meetings to identify policy leverage points and developing pilot programmes to test pooled demand models. Despite these limitations, this study offers a roadmap and framework for targeted interventions to improve airway equipment availability and access.

Addressing airway equipment shortages in SSA requires systemic reforms and multilevel interventions. By strengthening local manufacturing, reforming procurement policies and fostering collaboration among key stakeholders, sustainable solutions can be developed to ensure equitable access to life-saving airway equipment. This research highlights critical leverage points for intervention, offering a foundation for future policy and procurement improvements.

## Conclusion

This study provides a detailed analysis of airway equipment procurement in SSA, identifying key challenges and opportunities for strengthening regional healthcare delivery. Through interviews with otolaryngologists, manufacturers and policymakers, key themes and challenges emerged around manufacturing capacity, supply chains, cost and regulatory policy. Despite these obstacles, opportunities exist to improve access through local manufacturing, public–private partnerships and streamlined procurement systems. Strengthening communication between stakeholders is essential for building a sustainable, resilient and responsive infrastructure. By addressing barriers and implementing practical solutions, stakeholders can enhance surgical care capacity and promote health equity in the region.

## Appendix 1

**TABLE 1-A1:** Qualitative semi-structured interview guide.

Primary question	Probes and additional questions
What is your country or region of practice? Do you practice in private or public (government) or both?	-
What are the bottlenecks or greatest challenges of procuring airway-related equipment in your country or hospital?	Who are the main stakeholders in equipment procurement in your country? Who are the key stakeholders in government? What are the ways to target or approach these politicians?
	What, if any, are the barriers in airway equipment procurement? What is the main barrier (i.e. cost, policy, transport etc.)?
What equipment or materials related to airway management do you think should be prioritised?	-
What should be the next step in achieving airway equipment? Getting consistent access to airway equipment is challenging and requires input and processes from multiple bodies and organisations. This is a challenging problem. Given this complex situation, what should be prioritised or could be possible solutions to improving access to airway equipment?	What can stakeholders (medical supply companies, middlemen and hospital administrators) do to improve access to airway equipment?
	What information is needed to guide the next actionable step? Getting a stable system for equipment access is a challenge. What are the key action items that should be prioritised (at the hospital, national, public versus private sector etc.)?
Given the lack of equipment, what are the ways you work around these limitations? Donated or repurposed equipment could be one way around the limitations in equipment. What are your views about donated or repurposed equipment (assessing general acceptability)?	What are the barriers to receiving donations of equipment?
	What types of equipment would you be willing or want to accept?
	Would you be willing to accept USED equipment donations?
	Would you be willing to accept NEW (unused) equipment donations?
	Have you previously received unused equipment donations or supplies?
In addition to equipment, management of airways is complex. What are your thoughts on other aspects that should be prioritised when thinking about improving airway management for patients?	-
Optional:	
Other than airway equipment, are there other types of equipment that you feel have limited access within your country?	Are there different stakeholders involved in the procurement of these other forms of equipment?
	Are there different barriers to acquiring these other forms of equipment?

**TABLE 2-A1:** Participant demographics and data sources.

Participant category	Number of participants	Data collection modality	Geographic scope	Relevant expertise or role
Practising otolaryngologists and airway specialists in sub-Saharan Africa	13	Semi-structured in-person interviews; in-person focus groups; written responses	Uganda (*n* = 2), Tanzania (*n* = 1), Zimbabwe (*n* = 1), South Africa (*n* = 3), Kenya (*n* = 3), Rwanda (*n* = 1), Zambia (*n* = 1), Ghana (*n* = 1)	Subspecialty paediatric airway training; complex paediatric airway care in resource-limited settings
Manufacturing representatives from companies supplying SSA markets	3	Semi-structured virtual interviews	Olympus; Karl Storz; Stryker	Design, production, distribution and after-sales support of airway equipment
International policymakers	2	Semi-structured virtual interviews	World Health Organization (WHO)	Emergency health policy; procurement regulation; global health financing

Note: The table summarises the demographic characteristics of participants included in the study and the qualitative data collection methods used. The table illustrates how perspectives were captured across multiple stakeholder groups (including regional otolaryngologists, paediatric airway specialists, manufacturers and international policymakers) spanning diverse countries in sub-Saharan Africa and international organisations. Data were collected through a combination of semi-structured interviews, written responses and a focus group, allowing for in-depth exploration and mapping of systemic challenges and barriers within airway equipment procurement processes.

SSA, sub-Saharan Africa.

## References

[1] Meara JG, Leather AJ Hagander L, et al. Global surgery 2030: Evidence and solutions for achieving health, welfare, and economic development. Lancet;386(9993):569–624. 10.1016/S0140-6736(15)60160-X 25924834

[2] Nuss S, Patterson RH, Cahill GL, et al. Delphi method consensus on priority global otolaryngology-head and neck surgery conditions and procedures. Otolaryngol Head Neck Surg. 2022;167(4):669–677. 10.1177/01945998211073705 35077240

[3] Nuss S, Cahill GL, Limenh W, Wiedermann J. Developing consensus on priority pediatric otolaryngology-head and neck surgery conditions and procedures. Otolaryngol Head Neck Surg. 2023;169(2):374–381. 10.1177/01945998211073705 36939625

[4] Mock CN, Donkor P, Gawande A, et al. Essential surgery: Key messages from disease control priorities, 3rd edition. Lancet. 2015;385(9983):2209–2219. 10.1016/S0140-6736(15)60091-5 25662414 PMC7004823

[5] Miller FA, Young SB, Dobrow M, Shojania KG. Vulnerability of the medical product supply chain: The wake-up call of COVID-19. BMJ Qual Saf. 2021;30(4):331–335. 10.1136/bmjqs-2020-012133 33139342

[6] Patterson RH, Bangash AH, Zalaquett N, et al. 2025. Global barriers to otolaryngology care. JAMA Otolaryngol Head Neck Surg. 10.1001/jamaoto.2025.0573 PMC12022866 40272811

[7] Srinivasan T, Cherches A, Seguya A, et al. 2025. Essential equipment for baseline otolaryngology-head and neck surgery care: A global cross-sectional survey. Laryngoscope Investig Otolaryngol. 2025;10(1):e70078. 10.1002/lio2.70078 PMC11826442 39958942

[8] Kabagenyi F, Cherches AD, Patel NR, et al. Paediatric airway foreign-body removal equipment availability in sub-Saharan Africa. J Coll Med S Afr. 2024;2(1):45. 10.4102/jcmsa.v2i1.45 40949655 PMC12376285

[9] Melaku G. Foreign body aspiration in children: Experience from Ethiopia. East Afr Med J. 1996;73(7):459–462. 8918009

[10] Hofmeyr RH, McGuire JM, Marwick PM, et al. Assessment of continuous ventilation during tracheal dilatation using a novel, non-occlusive balloon in an ovine model. S Afr Journal of Anaesth Analg. 2020;26(5):245–249. 10.36303/SAJAA.2020.26.5.2383

[11] Nazir A, Vervoort D, Reddy CL. From the first mile to the last: Challenges of the global surgical supply chain. Am J Surg. 2021;222(4):709. 10.1016/j.amjsurg.2021.03.033 33775412 PMC7983454

[12] MDDI Online. How to prepare for new pricing rules and consolidation in China’s medical device distribution system [homepage on the Internet]. Medical Device and Diagnostic Industry; 2023. [cited 2025 Sep 01]. Available from: https://www.mddionline.com/business/how-to-prepare-for-new-pricing-rules-and-consolidation-in-china-s-medical-device-distribution-system

[13] Larson C, Burn R, Minnick-Sakal A, Douglas MOK, Kuritsky J. Strategies to reduce risks in ARV supply chains in the developing world. Glob Health Sci Pract. 2014;2(4):395–402. 10.9745/GHSP-D-14-00105 25611474 PMC4307856

[14] Alberti P, Kisa P. Paediatric surgery in Uganda: Current challenges and opportunities. Discov Health Syst. 2024;3(1):29. 10.1007/s44250-024-00076-8

[15] Bryce E, Fedatto M, Cunningham D. Providing paediatric surgery in low-resource countries. BMJ Paediatr Open. 2023;7(1):e001603. 10.1136/bmjpo-2022-001603 PMC9923288 36764702

